# Possible interplay between estrogen and the BAFF may modify thyroid activity in Graves’ disease

**DOI:** 10.1038/s41598-021-00903-5

**Published:** 2021-11-01

**Authors:** Chao-Wen Cheng, Wen-Fang Fang, Kam-Tsun Tang, Jiunn-Diann Lin

**Affiliations:** 1 Graduate Institute of Clinical Medicine, College of Medicine, Taipei Medical University, Taipei, 11031 Taiwan; 2 Traditional Herb Medicine Research Center, Taipei Medical University Hospital, Taipei Medical University, Taipei, 11031 Taiwan; 3 Cell Physiology and Molecular Image Research Center, Wan Fang Hospital, Taipei Medical University, Taipei, 11031 Taiwan; 4 Department of Family Medicine, Wan Fang Hospital, Taipei Medical University, Taipei, 11031 Taiwan; 5 Division of Endocrinology and Metabolism, Department of Internal Medicine, Taipei Veterans General Hospital, Taipei, 112 Taiwan; 6 Division of Endocrinology, Department of Internal Medicine, Shuang Ho Hospital, Taipei Medical University, 291 Jhongzheng Rd., Jhonghe District, New Taipei City, 23561 Taiwan; 7 Division of Endocrinology and Metabolism, Department of Internal Medicine, School of Medicine, College of Medicine, Taipei Medical University, Taipei, 11031 Taiwan

**Keywords:** Immunology, Biomarkers, Endocrinology

## Abstract

A link between sex hormones and B-cell activating factor (BAFF), a crucial immunoregulator of autoimmune thyroid disease (AITD), may exist. The study aimed to elucidate the role of estrogen (E2) in regulating BAFF in Graves' disease (GD). In clinical samples, serum BAFF levels were higher in women than in men in both the GD and control groups. serum BAFF levels were associated with thyroid-stimulating hormone receptor antibody levels and thyroid function only in women and not in men. *BAFF* transcripts in peripheral blood mononuclear cells were higher in women with GD than those in the control group. Among GD patients with the AA genotype of rs2893321, women had higher *BAFF* transcripts and protein levels than men. In the progression of a spontaneous autoimmune thyroiditis (SAT) murine model, NOD.H-2h4, serum free thyroxine and BAFF levels were higher in female than in male mice. Moreover, exogenous E2 treatment increased serum BAFF levels in male SAT mice. Meanwhile, female SAT mice exhibited higher thyroid *BAFF* transcripts levels than either the E2-treated or untreated male SAT mouse groups. Our results showed that E2 might be implicated in modulating BAFF expression, and support a possible mechanism for the higher incidence of AITD in women.

## Introduction

Graves' disease (GD) is one of the most prevalent tissue-specific autoimmune diseases (AIDs) in the world^1^. Different from other AIDs which destroy the target organs, GD is characterized by thyrocyte hyperplasia, thyroid gland hypertrophy, and upgraded thyroid function. According to serological laboratory analyses, patients with GD display elevated thyroid hormone, suppressed thyroid-stimulating hormone (TSH), and detectable TSH receptor (TSHR) antibody (TSHRAb) levels. It is well characterized that GD is pathogenically driven by an antibody-mediated immune reaction, which targets one of the important thyroid-specific proteins, the TSHR, and induces synthesis of the TSHRAb. The TSHRAb can be divided into two forms, stimulating and blocking type, by utilizing the functional cell-based assay^2^. The circulating stimulating type of TSHRAb works like a TSH agonist, can specifically bind to the TSHR, subsequently stimulates thyrocyte proliferation and hypertrophy, promotes the sodium-iodide symporter, thyroglobulin, and thyroperoxidase gene expressions, and ultimately enhances thyroid hormone production^3^. On the other hand, blocking type of TSHRAb exhibits no functional activity after binding to TSHR, and is regarded as a neutral form of antibody^4^.

B-Cell-activating factor (BAFF) is a potent stimulator of B-cell maturation and differentiation^5,6^. It was reported that the BAFF is expressed by many cell types, including monocytes, dendritic cells, neutrophils, stromal cells, epithelial cells, and lymphocytes^7^. *BAFF* transgenic mice showed increased B-cell numbers, upregulated immunoglobulin, and development of pathogenic features similar to those of systemic lupus erythematosus (SLE)^8^. Upregulated BAFF activity was found to contribute to the occurrence of multiple AIDs^9–11^, including GD. Fabris et al. and Vannucchi et al. demonstrated that plasma BAFF levels were enhanced in patients with GD^12,13^. In addition to overexpression of the BAFF protein in GD, Vannucchi et al. also found that serum BAFF protein levels declined in response to immunomodulatory treatment^13^. In our recent study, we also found that serum BAFF levels were higher in GD patients, and BAFF protein levels could modulate clinical phenotypes of GD at the baseline^14^. In addition, we also observed that rs2893321, a *BAFF* intronic single-nucleotide polymorphism (SNP), affected susceptibility to GD^15^. All those studies highlighted the key role of the BAFF in the pathogenesis of GD. On the other hand, our previous studies demonstrated that associations of the rs2893321 SNP and serum BAFF levels with the occurrence of GD and clinical features of GD at the baseline were more remarkable in women than in men, which highly suggests there is a possible interplay between BAFF and sex steroids in the pathogenesis of GD.

The current study aimed to elucidate sexually dimorphic patterns in the influence of serum BAFF levels in GD, and the possible modulatory activity of estrogen (E2). We analyzed relationships among BAFF messenger (m)RNA, serum BAFF levels, and clinical characteristics in clinical specimens of both genders and in a spontaneous autoimmune thyroiditis (SAT) mouse strain, NOD.H-2h4. In addition, E2 was exogenously administered to male SAT mice to evaluate the modulatory activity of E2 on BAFF production.

## Results

### Serum BAFF protein levels were particularly associated with thyroid function and TSHRAb titers in female GD patients

Demographic data of the GD (*n* = 237) and control (*n* = 183) groups are shown in Table 1. The numbers of patients with a family history (FH) of thyroid disease and smoking habit were higher in the GD group than in the control group. There was no difference in age or the percentage of women between the GD and control groups. On the other hand, clinical parameters between women and men in GD were also compared. Men had a higher percentage with a smoking habit than did women, while there were no significant differences in age, FH percentage, or FT4 and TSHRAb levels at enrollment between men and women (data shown in Supplementary Table 2).

**Table 1:** Demographic characteristics of Graves’ disease (GD) patients and a healthy control group at the time of sample collection.

	Control	GD	*p* value
	*N* = 183	*N* = 237	
Age (years)	42.0 ± 9.3	42.2 ± 12.9	0.856
Sex (female %)	58.7	66.4	0.103
Smoking (%)	15.2	24.7	0.018
Family history of thyroid disease (%)	4.4	28.6	< 0.001

*p* < 0.05 indicates statistical significance.

**Figure 1:**
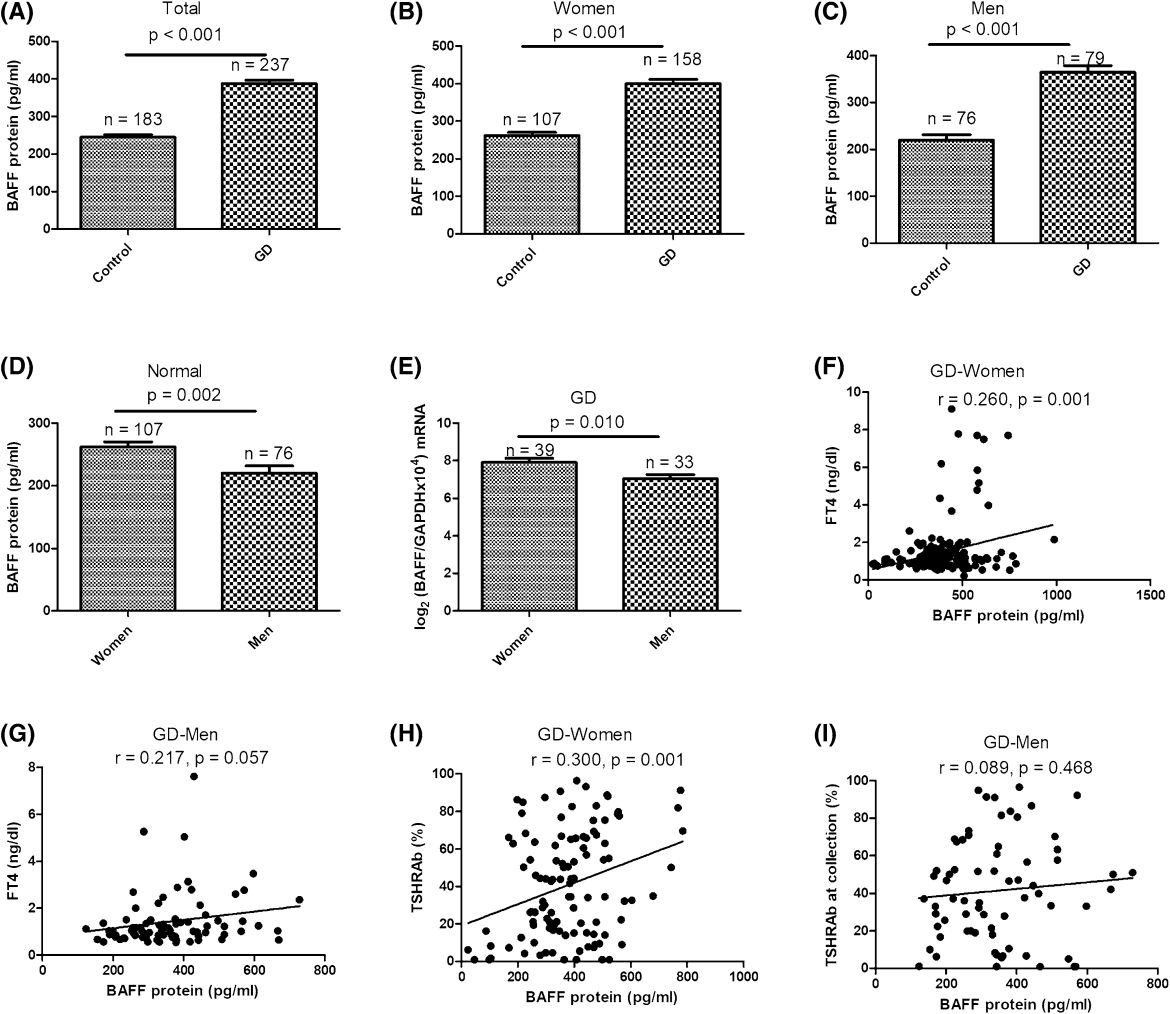
Comparisons of serum B-cell-activating factor (BAFF) protein levels between Graves' disease (GD) and control subjects in (**A**) all, (**B**) female, and (**C**) male subjects, BAFF protein levels between women and men in normal subjects (**D**) and GD patients (**D**,**E**), and associations of BAFF protein levels with free thyroxine (FT4) in women (**F**) and men (**G**) with GD, and thyroid-stimulating hormone receptor antibody (TSHRAb) titers in women (**H**) and men (**I**) with GD. Data are expressed as the mean ± standard error. *p* < 0.05 indicates statistical significance.

Serum BAFF levels were significantly higher in the GD group than in the control group (*p* < 0.001, Fig. 1A). In addition, we determined serum BAFF levels in both sexes, and found that BAFF protein levels were also higher in the GD group than in the control group in both women (*p* < 0.001; Fig. 1B) and men (*p* < 0.001; Fig. 1C). Women had higher serum BAFF levels than men in both healthy controls (Fig. 1D) and GD patients (Fig. 1E).

In analyzing associations of serum BAFF levels with thyroid function in GD, FT4 levels were also significantly correlated with BAFF protein levels in women (*r* = 0.260, *p* < 0.001; Fig. 1F) but not in men (*r* = 0.217, *p* = 0.057; Fig. 1G). In addition, a significant association of serum BAFF levels with TSHRAb levels was observed in women (*r* = 0.300, *p* = 0.001; Fig. 1H) but not in men (*r* = 0.089, *p* = 0.468; Fig. 1I).

### *BAFF* transcripts in PBMCs were correlated with serum BAFF levels in GD patients

**Figure 2:**
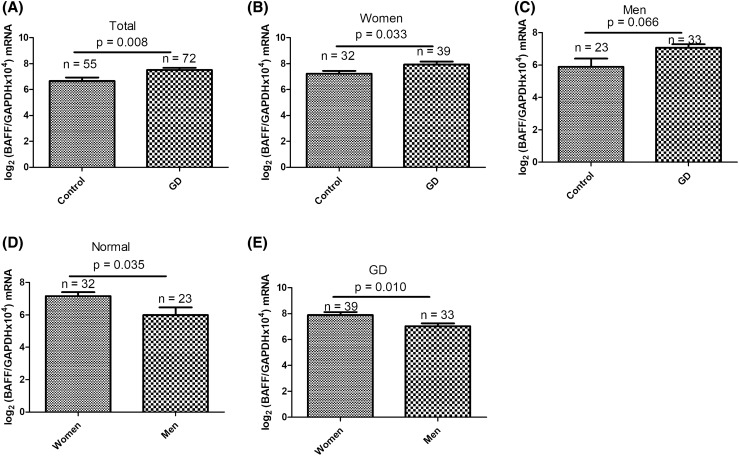
Comparison of *B-cell-activating factor (BAFF)* mRNA levels from peripheral blood mononuclear cells (PBMCs) between Graves' disease (GD) patients and healthy controls and in (**A**) all, (**B**) female, and (**C**) male subjects, and differences in BAFF mRNA levels between women and men in normal subjects (**D**) and GD patients (**E**).

Thereafter, freshly collected PBMC samples (55 controls and 72 GD patients) were applied to assess differential *BAFF* transcripts in PBMCs (demographic data are presented in Supplementary Table 3). Along with serum protein levels, *BAFF* transcripts in PBMCs were higher in patients with GD than that in control subjects (*p* = 0.008; Fig. 2A). *BAFF* transcripts were higher in the GD group than in the control group in women (*p* = 0.033; Fig. 2B). However, in men, although *BAFF* transcript levels were also higher in GD patients compared to control subjects, it did not reach statistical significance (*p* = 0.066; Fig. 2C). Women had higher *BAFF* mRNA expression levels than men in both healthy controls (*p* = 0.035; Fig. 2D) and GD patients (*p* = 0.010; Fig. 2E).

**Figure 3:**
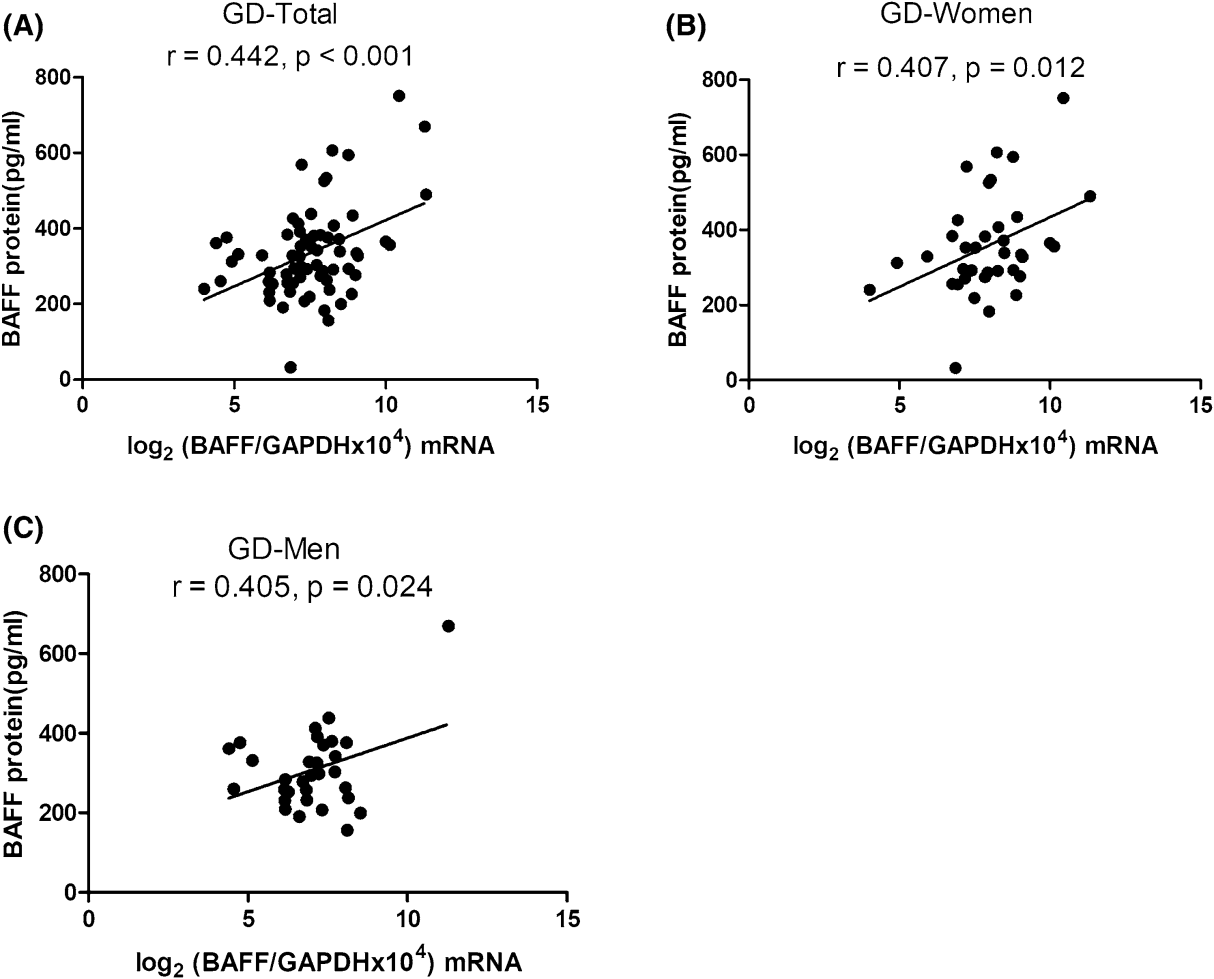
Correlations between serum B-cell-activating factor (BAFF) levels and *BAFF* mRNA levels in (**A**) all, (**B**) female, and (**C**) male Graves’s disease (GD) patients. *p* < 0.05 indicates statistical significance.

There was a significant association of *BAFF* transcript levels in PBMCs with serum BAFF protein levels in patients with GD (*r* = 0.442, *p* < 0.001; Fig. 3A). At the same time, we also observed that there were significant correlations between *BAFF* transcripts and BAFF protein levels in both women (*r* = 0.407, *p* = 0.012; Fig. 3B) and men with GD (*r* = 0.405, *p* = 0.024; Fig. 3C).

### The AA genotype of rs289332 tended to increase susceptibility to higher *BAFF* transcript and serum protein levels in female GD patients

**Figure 4:**
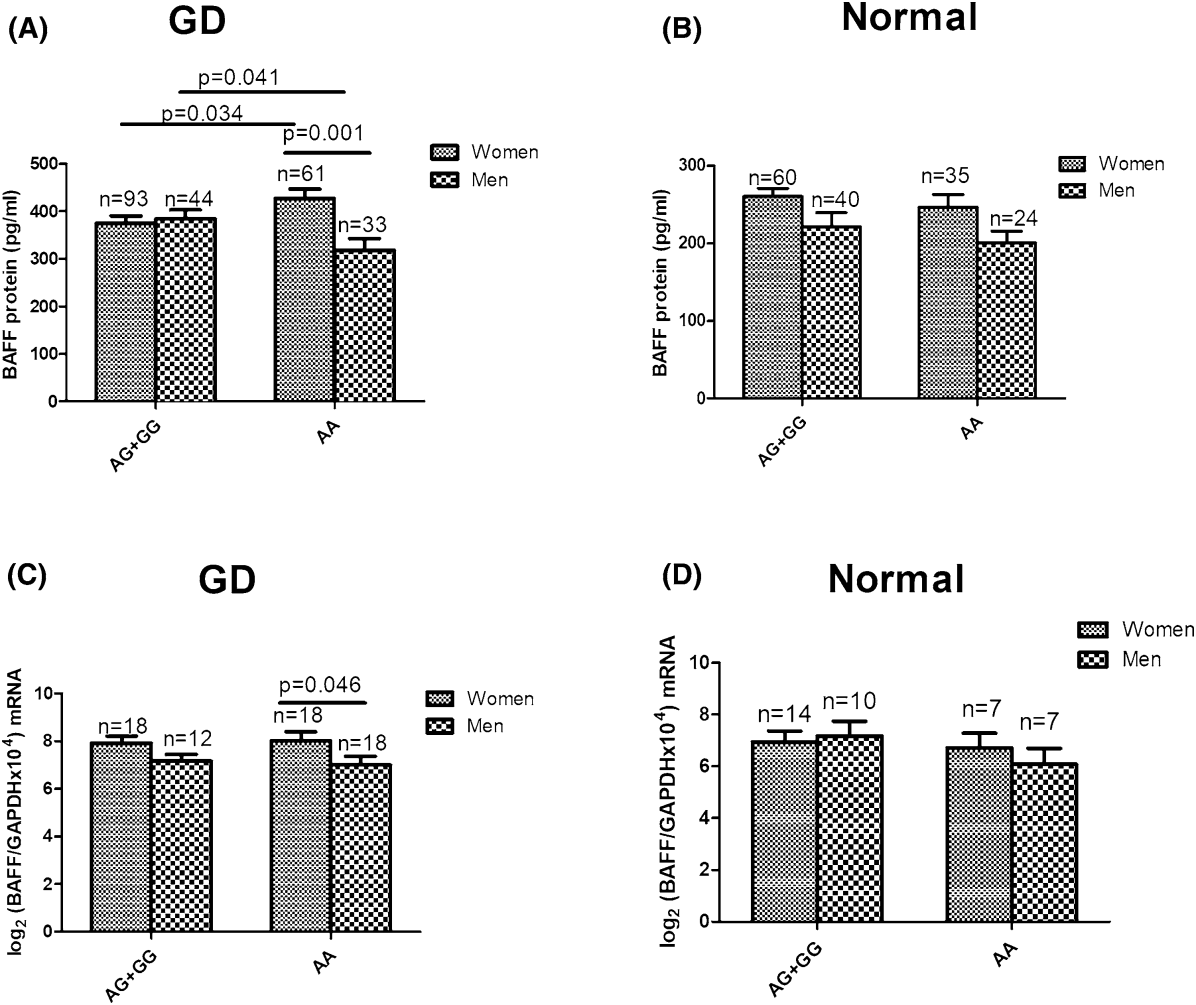
Comparisons of B-cell-activating factor (BAFF) protein (**A**,**B**) and *BAFF* mRNA levels (**C**,**D**) between women and men with different genotypes of rs2893321 in Graves' disease (GD) and healthy controls.

In analyzing genetic variants of rs2893321, there was no significant difference in BAFF protein levels between women and men with the AG + GG genotype in either the GD (*p* = 0.712; Fig. 4A) or control groups (*p* = 0.069; Fig. 4B). However, among those with the AA genotype, female GD patients had higher BAFF protein levels than male GD patients (*p* = 0.001; Fig. 4A), while there was no significant difference in BAFF protein levels between female and male healthy controls (*p* = 0.056; Fig. 4B). At the same time, among subjects with the AG + GG genotype, there was no significant difference in *BAFF* transcripts between women and men in the GD (*p* = 0.095; Fig. 4C) or control groups (*p* = 0746; Fig. 4D). Meanwhile, among GD patients with the AA genotype, women had higher *BAFF* transcript levels than men (*p* = 0.046; Fig. 4C), but no significant difference existed between normal female and male subjects (*p* = 0.450; Fig. 4D).

### Thyroid function and BAFF protein levels were higher in female SAT mice

**Figure 5:**
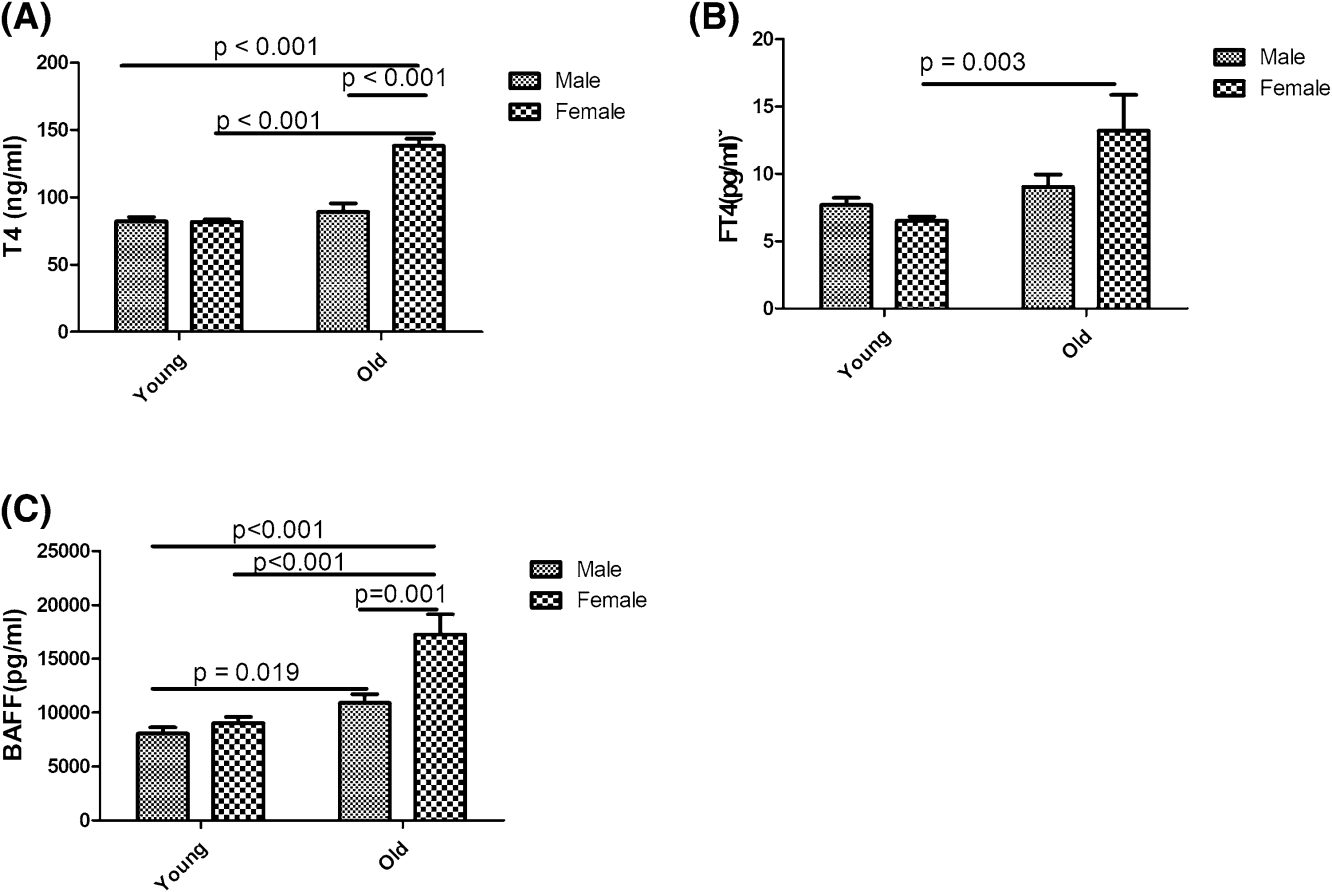
Total thyroxine (T4) (**A**), free thyroxine (FT4) (**B**), and B-cell-activating factor (BAFF) protein levels (**C**) among young male, young female, old male, and old female mouse groups.* p < 0.05.

In addition to clinical subjects, we also examined the effects of gender differences in thyroid function and levels of BAFF protein in a SAT mouse model. All of the SAT mice exhibited normal thyroid features at a younger age (3 ~ 6 weeks), while they presented with various degrees of thyroiditis at an older age (16 ~ 18 weeks) in both males and females (data not shown). As shown in Fig. 5A, T4 levels showed no difference between different genders at the younger age. There was no difference in T4 levels between younger- and older-aged male mice. On the other hand, female SAT mice showed significantly higher T4 levels than younger female mice and older male mice. On the other hand, old female mice had higher FT4 levels than young female mice (Fig. 5B). Although both female and male SAT mice exhibited higher levels of serum BAFF protein at an older age than at a younger age, female mice had significantly higher levels than male mice, and results are shown in Fig. 5C.

### E2 increased BAFF protein levels and thyroid function in male SAT mice

**Figure 6:**
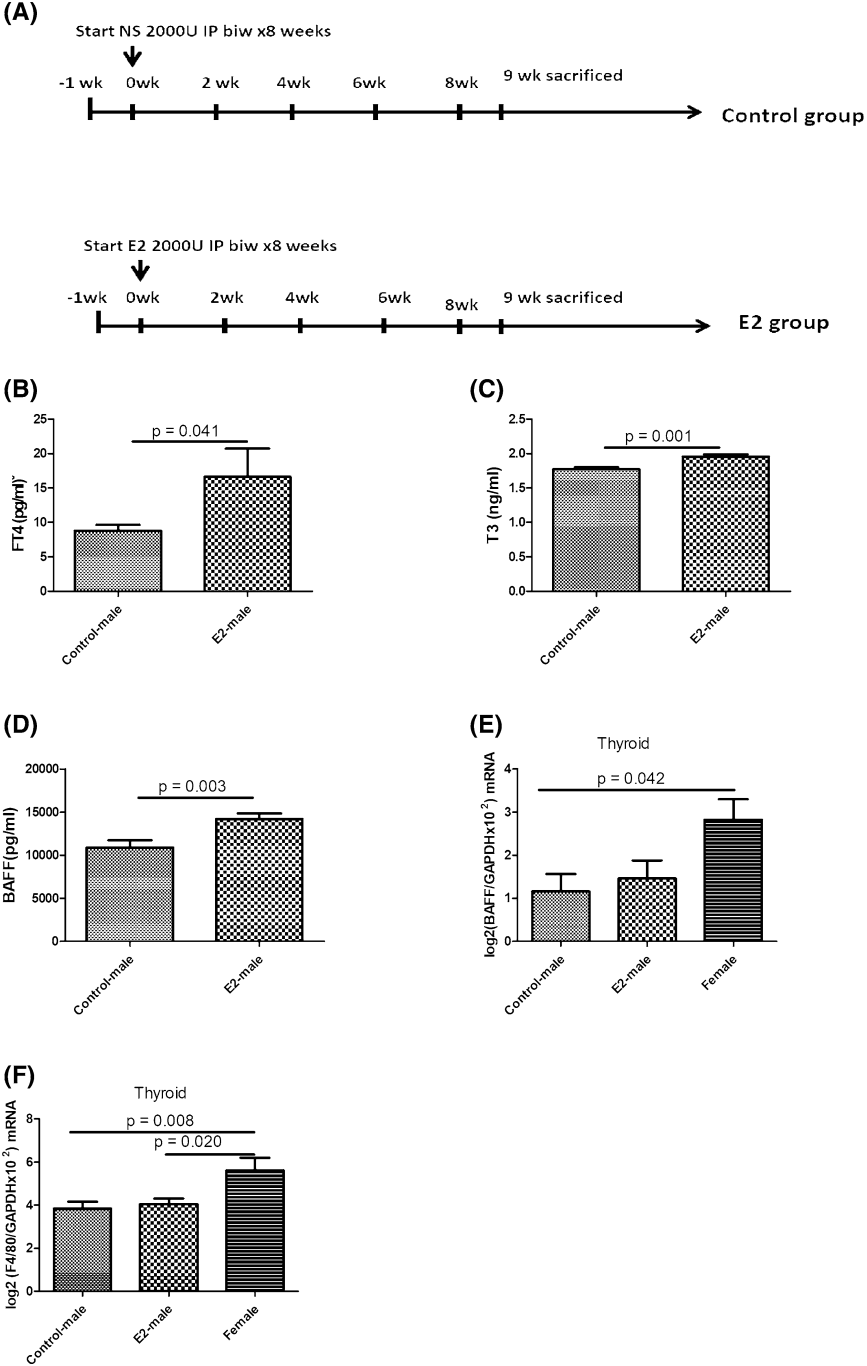
Protocol of estrogen (E2) treatment in NOD.H-2h4 mice (**A**), and (**B**) the free thyroxine (FT4), (**C**) triiodothyronine (T3), and (**D**) B-cell-activating factor (BAFF) protein levels between control-male and E2-treated mice, and (**E**) *BAFF*, and (**F**) *F4/80* mRNA levels among control-male, E2-treated male, and female mouse groups.

In order to elucidate the impact of sex hormone on BAFF protein production, we administered E2 to SAT male mice following the procedure in Fig. 6A. As shown in Fig. 6B,C, treatment with E2 increased FT4 (*p* = 0.041) and T3 levels (*p* = 0.001) compared to the control group. In addition, E2-treated mice showed higher plasma BAFF protein levels than control mice (*p* = 0.003, Fig. 6D). However, E2-treated and untreated male SAT mice both showed lower thyroid BAFF and F4/80 mRNA levels than female SAT mice (Fig. 6E,F).

## Discussion

In the study, we first showed that *BAFF* transcripts from PBMCs were upregulated and correlated with serum BAFF levels in GD patients compared to healthy controls, which implied that *BAFF* mRNA in PBMCs could be the origin of serum BAFF protein synthesis in GD. At the same time, we detected that BAFF protein and mRNA levels were higher in women than in men in both GD patients and normal controls. In addition, in GD patients with the rs2893321 AA genotype, we observed that women had higher *BAFF* mRNA and protein levels than did men. On the other hand, in the progression of SAT mouse model, female mice exhibited higher thyroid function and BAFF protein levels, but these were not seen in male mice. In addition, exogenously administered E2 induced an increase in thyroid function and a rise in serum BAFF levels in male SAT mice. From clinical observations to animal models, these lines of evidence all supported that the sex steroid, E2, was linked to the increase in serum BAFF protein levels, which contributed to the pathogenesis of AITD.

In our previous study, we showed significant correlations of serum BAFF levels with FT4 and TSHRAb levels at the baseline, which might lead to a possible bias due to different time points of collecting blood samples for measuring the BAFF, thyroid function, and TSHRAb^14^. To further precisely verify how the BAFF protein controls clinical parameters, we enrolled more subjects and compared serum BAFF levels and TSHRAb levels at the time of collection, not at the baseline, and associations of BAFF protein with thyroid function and TSHRAb levels were still present. Moreover, associations of BAFF protein levels with thyroid function and TSHRAb levels consistently only existed in women and not in men. On the other hand, we also observed that women had higher BAFF protein levels compared to men in both GD patients and normal controls. Both of the above findings indicated that sex hormones, possibly E2, could modulate BAFF translation and also be implicated in the BAFF protein's control of thyroid hormone production and TSHRAb expression after disease onset.

In the pathogenesis of GD, Campi et al. reported that BAFF gene expression in the thyroid gland could be a locally important source of the BAFF protein pool^16^. Meanwhile, in the current study, we first found that *BAFF* mRNA derived from PBMCs could be another significant origin of serum BAFF protein production in GD patients. These findings are compatible with several previous research studies on SLE and multiple myelomas, which demonstrated increased *BAFF* transcripts in PBMCs after disease onset compared to normal controls^17–20^. Interestingly, there was only a moderate correlation between *BAFF* transcripts in PBMCs and serum BAFF protein levels in GD (*r* = 0.442, *p* < 0.001), which was not a surprising finding. As mentioned above, *BAFF* gene expression is present in a broad range of cell types and tissues, and BAFF expression from PBMCs only accounts for a portion of the serum BAFF protein production system^16,17,21^. In addition, we found that women had higher *BAFF* mRNA levels than men in both the GD and control groups. These observations also reflect a possible effect of E2 in modulating the *BAFF* transcription process.

In GD, women with the AA genotype of rs2893321 had higher *BAFF* mRNA and protein levels compared to men, while associations of gender with *BAFF* mRNA and protein levels were absent in those with the AG + GG genotype. These findings imply that this non-coding SNP of rs2893321 could be engaged in controlling *BAFF* mRNA and protein production in GD, and the genetic influence on *BAFF* gene expression of rs2893321 is likely regulated by E2. Apart from a possible linkage to the coding region^22^, the non-coding SNP could elicit biological functions by way of influencing the processes of mRNA formation and protein translation, which are reflected by either alterations in mRNA and protein levels or the formation of splicing variants and protein isoforms^23–25^. On the other hand, the actual mechanism of E2 in regulating SNP function is unclear. Liu et al. and Hayase et al. suggested that sex steroids can act as transcription factors to bind to the locus close to the genetic variant to directly regulate gene expression, which might partially explain our observations^26,27^. However, different from results in GD patients, *BAFF* mRNA and protein levels did not differ between normal women and men, which implied that E2 could play a limited role in modifying the genetic influence on *BAFF* mRNA and protein production in normal subjects. The reason for this discordance between GD and healthy subjects is unknown. It might be attributed to some feedback regulatory mechanism in normal subjects, which offsets the influence of sex steroids of modulating the genetic effect on BAFF protein production. Additional studies are needed to clarify these discrepant results.

The NOD.H-2h4 mouse strain was characterized to have features of a high incidence rate (~ 50%) of the spontaneous development of thyroiditis^28^, while in the presence of NaI in drinking water, the incidence of SAT increased to near 100%^29^. However, in order to specifically emphasize the effects of gender differences, animals applied in the current study were not given NaI in drinking water. Our data showed that female SAT mice, similar to clinical observations, showed both higher FT4 and serum BAFF protein levels in full-blown status. In addition, E2-treated mice had higher FT4 and T3 levels compared to those in the control group. It is established that E2 can increase thyroid-binding globulin (TBG) level and subsequently increase total T3 and T4 level but without altering free form of thyroid hormone^30^. In the study, serum FT4 level was increased in the progression of female SAT model, and E2-treated male SAT mice, which indicated that the thyroid function was actually increased after E2 treatment, but was not attributed to the increased TBG level^30,31^. In addition, we demonstrated that E2-treated mice had higher BAFF protein levels compared to the control group. Data from the animal experiment support our clinical results, which showed that sex steroids could play roles in directly influencing BAFF production, and induce an elevation in thyroid function through an uncertain mechanism. However, lower thyroid *BAFF* and *F4/80* mRNA levels were found in both E2-treated and untreated male mice than in aged female mice. The discontinuous exposure of exogenous E2 (twice a week) in male mice might be unable to achieve comparable physiological E2 concentrations as in female mice. In addition to regulating *BAFF* mRNA expression, E2 might either enhance membrane-bound BAFF protein cleavage and release or inhibit BAFF protein degradation^32–34^, which would lead to the accumulation of BAFF proteins in plasma.

In conclusion, in the present study, we found a possible role of E2 in regulating BAFF expression and subsequently shape thyroid activity and TSHRAb levels in GD. Furthermore, we confirmed the E2 could increase thyroid hormone levels and upregulated BAFF protein levels in a SAT mouse model. To the best of our knowledge, this is the first study to demonstrate the potential of E2 to modulate BAFF expression in GD. However, our study has several limitations that should be addressed. First, despite demonstrating associations of the AA genotype with *BAFF* transcripts and proteins in women with GD, which implied E2 could possibly exert a role in regulating r2893321 in modulating BAFF expression, we did not confirm that E2 amends the genetic effect of a *BAFF* SNP of rs2893321 in modulating *BAFF* transcription and translation by using either in vivo or in vitro experiments. The limited sample size and different subject numbers between women and men in the GD and control groups could lead to possible bias in the results. Treatment of primary cell cultures of the AA genotype of rs2893321 from PBMCs of GD patients and healthy controls with E2 could further clarify the role of E2 in controlling BAFF synthesis. Second, different from GD, which is an antibody-mediated AITD, the NOD.H-2h4 mouse strain is a mainly T-cell driven autoimmune thyroiditis animal model. So far, no appropriate animal model fully mimicking GD in the clinical setting was established successfully, therefore, we can only utilize this SAT mouse model to verify the clinical findings in GD in this study. Accordingly, the findings from the SAT animal experiments may not be able to entirely reflect the actual effect of E2 on BAFF in the GD. Moreover, the influence of E2 on either direct TSHRAb production or the capacity of BAFF in regulating TSHRAb and thyroid function was unable to be evaluated in our clinical results, and more researches focusing on BAFF expression in a suitable GD animal model might further support our findings.

## Materials and methods

### Clinical study

#### Samples

This research was comprised of two studies: the first study recruited 290 participants (165 GD patients and 121 healthy controls) from the Division of Endocrinology, Department of Internal Medicine, and the Health Screening Center of Shuang-Ho Hospital (New Taipei City, Taiwan) from January 2013 to September 2014 (201404091). The second study enrolled 134 subjects (72 GD patients and 62 healthy controls) from the Division of Endocrinology and Metabolism and Healthy Screening Center of Shuang Ho Hospital from May 2016 to May 2018 (N201602050). In total, blood specimens of 237 patients with GD, aged more than 20 years, and blood samples of 183 patients without AITD or other AIDs and aged more than 20 years were obtained. Patients with AITD and healthy controls were excluded if they were aged less than 20 years, pregnant, or alcoholic, or had a history of drug intoxication. The study protocol was approved by the Joint Institutional Review Board of Taipei Medical University, and all experiments were performed in accordance with relevant named guidelines and regulations. All participants provided written informed consent prior to participation.

GD was diagnosed if one of the following criteria was met: (1) the presence of a low TSH level, a normal or high free thyroxine (FT4) level, and TSHRAbs; (2) the presence of thyrotoxicosis without TSHRAbs but increased or normal diffuse thyroid uptake of I^131^; or (3) a proven diagnosis by another hospital, as indicated by medical records.

### Laboratory analyses

Serum FT4 and TSH levels were determined with an electrochemiluminescence immunoassay method using commercial Roche Elecsys reagent kits (Roche Diagnostica, Switzerland). The normal range of FT4 is 0.93 ~ 1.7 ng/dL (with an intra-assay coefficient of variation [CV] of < 2.0% and an interassay CV of < 4.8%) and that of TSH is 0.27 ~ 4.20 μIU/mL (with an intra-assay CV of < 3.0% and an interassay CV of < 7.2%).

Serum TSHRAb levels were quantified through a radioimmunoassay method using a commercial TSHRAb-coated tube kit (R.S.R., Cardiff, UK). Data are expressed as the percentage of blocking of I^125^-labeled TSH binding to the TSH receptor coated onto a test tube^35^. A value of > 15% was considered positive.

BAFF levels in serum and supernatants of cell cultures were determined using a commercial enzyme-linked immunosorbent assay (ELISA) kit (R&D Systems, Minneapolis, MN, USA) according to the manufacturer’s protocol. Serum samples were diluted 1:threefold. Results are expressed as picograms BAFF per milliliter (pg/ml).

#### Genotyping

Genomic DNA was isolated from 3 ml of EDTA-containing whole-blood samples using a commercial DNA blood kit (Geneaid, Taiwan). Genotyping was done by a polymerase chain reaction (PCR)-restriction fragment length polymorphism (RFLP) method. PCR amplification was performed using 1 μl DNA, 1 μl primer, 10 μl Tag PCR MasterMix (Genomics Biosci & Tech, Taiwan), and 7 μl H_2_O. PCR conditions for rs2893321 were as follows: 30 cycles of denaturation at 94 °C for 30 s, annealing at 55 °C for 30 s, and extension at 72 °C for 30 s, with a final 7-min extension at 72 °C. Primer sequences are shown in Supplementary Table 1. PCR products were incubated with the Ase I restriction enzyme (New England Biolabs, Beverly, MA, USA) at 37 °C for 4 h. After incubation, DNA fragments were detected by electrophoresis with a 3% agarose gel. Primers and restriction enzymes for the SNP were as described in a previous report^15^ Approximately 10% of unrelated samples were subjected to repeat genotyping to exclude digestion errors, and no genotyping error was found. In addition, several PCR products were directly sequenced for quality control.

### Animal study

#### Preparation of mice

Briefly, 25 male NOD.H-2h4 mice were bred and obtained from the National Laboratory Animal Breeding and Research Center (NLABRC; Tainan, Taiwan). All mice were 8 ~ 10 weeks old at the beginning of the experiments^29^. Water-soluble β estradiol (E4389; Sigma, St. Louis, MO, USA) was subcutaneously administered to 11 mice (4 μg/mouse) twice a week for 8 weeks^36^. Another 14 mice were given the same volume of normal saline (NS) injected twice a week for 8 weeks. These mice were killed in the 8^th^ week, and the thyroid gland was removed to examine pathological features and conduct immunological analyses. FT4, T3, T4, and BAFF levels were measured in each mouse by commercial mouse ELISA kits.

Additionally, another 5 female and 5 male mice at 3 weeks old, 5 female and 7 male mice at 6 weeks old, and 7 female mice at 16 ~ 18 weeks old received from the NLABRC were directly sacrificed for reference. Young mice were defined as younger than 6 weeks, and old mice were older than 16 weeks. Therefore, in combination with E2-untreated male mice, we divided these mice into four groups, of young male, young female, old male, and old female groups. The protocol and procedures employed were ethically reviewed and approved by the Institutional Animal Care and Use Committee of Taipei Medical University (LAC-2019–0096) and all methods were performed in accordance with relevant guidelines and regulations, and all authors complied with ARRIVE guidelines.

### Mouse FT4, T4, T3, and BAFF measurements (ELISA):

The microplate provided in the ELISA kit was pre-coated with an antibody specific to FT4 (Elabscience Biotechnology, Houston, USA) or T3/T4 (CUSABIO Biotech, Wuhan, China). A standard or sample was added to the appropriate microtiter plate well with biotin-conjugated FT4/T3/T4. A competitive inhibition reaction was launched between FT4/T3/T4 and biotin-conjugated FT4/T3/T4 with the pre-coated antibody specific to FT4/T3/T4. After washing, avidin-conjugated horseradish peroxidase was added to the wells. A substrate solution was added to the wells, and the color was allowed to develop which was correlated with the amount of FT4/T3/T4 in the sample. Color development was stopped, and the intensity of the color was measured at OD 450 nm. Mouse plasma BAFF was also measured with an ELISA commercial kit (R&D Systems, Minneapolis, MN, USA)^37^.

### Purified RNA and first-strand complementary (c)DNA preparation

Peripheral blood mononuclear cell (PBMC) RNA was isolated from blood samples using a commercial kit (Quick-RNA™ Whole Blood, Zymogen Research, city?, ST?, USA). Thyroid tissue RNA was extracted with a GENEZol™ TriRNA Pure Kit (Geneaid, New Taipei City, Taiwan). RNA was dissolved in 15 µL of nuclease-free water, and purified RNA was stored at − 80 °C until further analysis. cDNA of purified RNA was prepared using a commercial cDNA synthesis kit (GoScript™, Promega, Madison, WI, USA). Reverse transcription was performed at 42 °C for 60 min and stopped by heating the mixture to 70 °C for 5 min. First-strand cDNA was stored at − 20 °C until experiments were conducted.

### Real-time quantitative polymerase chain reaction (qPCR)

For the real-time qPCR analysis, 300 ng of cDNA was added to 12 µL of buffer containing 10 µL of SYBR green reagent and 0.6 µL each of the forward and reverse primers (10 µM) in a total volume of 20 µL. The mixtures were amplified in a real-time PCR instrument (Biometra) under the following conditions: initial step at 50 °C for 2 min, followed by 95 °C for 10 s and 40 cycles of melting at 95 °C for 10 s, annealing at 55 °C for 36 s, and extension at 72 °C for 30 s. The quantity of each product was measured using the formula 2^−△Ct^, △meanCt = meanCt (target gene) − meanCt (GAPDH). The meanCt (*BAFF*) and meanCt (*GAPDH*) respectively indicate the average of Ct of *BAFF* and *GAPDH* in all experiments. Primer sequences of *BAFF*, F4/80, and GAPDH are shown in Supplementary Table 1.

### Statistical analysis

All statistical analyses were performed using SPSS software, vers. 13.0 for Windows (SPSS, Chicago, IL, USA). Quantitative values are presented as the mean ± standard deviation (SD) or mean ± standard error (SE). Because *BAFF* mRNA in the human study, and T4, FT4, BAFF protein, and *BAFF* mRNA in the animal study were shown to have a right-skewed distribution, they were log-transformed except for human and mouse *BAFF* mRNA which were log2-transformed. An independent *t*-test was used to compare differences in demographic data and different variables between two groups. Pearson's correlation was performed to assess relationships of FT4, TSHRAb, and log_2_*BAFF* mRNA with BAFF protein levels. Furthermore, a χ^2^ test or Fisher’s exact test was used to assess differences in categorical data between two groups. In the animal study, a one-way analysis of variance (ANOVA) was used to compare differences in FT4, T4, T3, and BAFF protein among the young male, young female, old male, and old female mouse groups, and was also used to compare differences in thyroid log_2_*BAFF* mRNA levels among the E2-treated male, control male, and female mouse groups. Tukey's test was used for post-hoc examinations. All statistical tests were two-sided, and a *p* value of < 0.05 was considered significant.

## Supplementary Information

Supplementary Information.

## Abbreviations

AID: Autoimmune disease
AITD: Autoimmune thyroid disease
BAFF: B-cell-activating factor
E2: Estrogen
GD: Graves’ disease
HT: Hashimoto’s thyroiditis
T4: Thyroxine
TSH: Thyroid-stimulating hormone
TSHR: Thyroid-stimulating hormone receptor
TSHRAb: Thyroid-stimulating hormone receptor antibody
