# Supplementary material for: Possible interplay between estrogen and the BAFF may modify thyroid activity in Graves’ disease
Source: Sci Rep. 2021 Nov 1;11:21350. doi: 10.1038/s41598-021-00903-5 (PMC8560878; doi:10.1038/s41598-021-00903-5)
Supplement: Supplementary file 1 — Supplementary Information. [file 41598_2021_903_MOESM1_ESM.docx]

Supplementary Table 1. Primer sequences of B-cell activating factor (BAFF) and glyceraldehyde-3-phosphate dehydrogenase (GAPDH)

| Name |  | Sequence 5’🡪3’ |
| --- | --- | --- |
| BAFF | Forward | CTATACAAAAAGGATCTTACAC |
|  | Reverse | GTCCCATGGCGTAGGTCTTA |
| GAPDH | Forward | CACCATCTTCCAGGAGCGAG |
|  | Reverse | TCACGCCACAGTTTCCCGGA |
| rs2893321 | Forward | TTTTTCGTTGGACTTGGTCA |
|  | Reverse | CAACCCAAATCCAGAATCCT |
| mBAFF | Forward | CAACGGAGACGACACCTTCT |
|  | Reverse | CTCATCCGGTCTTTCTGAGC |
| mGAPDH | Forward | CATCACTGCCACCCAGAAGACTG |
|  | Reverse | ATGCCAGTGAGCTTCCCGTTCAG |

BAFF, human B-cell activating factor;  GAPDH, glyceraldehyde-3-phosphate dehydrogenase

mBAFF, mouse BAFF, mGAPDH, mouse GAPDH

Supplementary Table 2. The comparisons of demographic characteristics, free thyroxine (FT4) and thyroid-stimulating hormone receptor antibody (TSHRAb) between women and men in Graves’ disease

|  | Women | Men | *p* value |
| --- | --- | --- | --- |
|  | *N* = 158 | *N* = 79 |  |
| Age (years) | 42.2 ± 12.9 | 40.6 ± 10.4 | 0.307 |
| Smoking (%) | 9.4 | 55.0 | < 0.001 |
| Family history of thyroid disease (%) | 28.4 | 28.9 | 1.000 |
| FT4 (ng/dl) | 1.49 ± 1.47 | 1.43 ± 1.14 | 0.737 |
| TSHRAb (%) | 41.0 ± 28.3 | 41.5 ± 27.5 | 0.914 |

FT4, free thyroxine; thyroid-stimulating hormone, TSHRAb, thyroid-stimulating hormone receptor antibody;  *p* < 0.05 indicates statistical significance.

Supplementary Table 3. Demographic data of 72 Graves’ disease (GD) patients and 55 subjects having peripheral blood mononuclear cells mRNA obtained at the time of sample collection

|  | Control | GD | *p* value |
| --- | --- | --- | --- |
|  | *N* = 55 | *N* = 72 |  |
| Age (years) | 37.3 ± 9.0 | 41.7 ± 11.4 | 0.019 |
| Sex (female %) | 58.2 | 54.2 | 0.652 |
| Smoking (%) | 10.9 | 26.4 | 0.030 |
| Family history of thyroid disease (%) | 5.5 | 26.4 | 0.002 |

*p* < 0.05 indicates statistical significance.
